# Periodic paralysis with normokalemia in a patient with hyperthyroidism: A case report

**DOI:** 10.1097/MD.0000000000013256

**Published:** 2018-11-16

**Authors:** Pin-Han Wang, Kuan-Ting Liu, Yen-Hung Wu, I-Jeng Yeh

**Affiliations:** a Department of Emergency Medicine, Kaohsiung Medical University Hospital; b School of Medicine, College of Medicine, Kaohsiung Medical University, Kaohsiung, Taiwan.

**Keywords:** hyperthyroidism, normokalemia, periodic paralysis

## Abstract

**Rationale:**

Thyrotoxic periodic paralysis is characterized by a sudden onset of hypokalemia and paralysis. This condition mainly affects the lower extremities and is secondary to thyrotoxicosis. The underlying hyperthyroidism is often subtle without typical symptoms such as palpitations, tremors, anxiety, and weight loss; this causes a difficulty in early diagnosis. Here, we reported a case of periodic paralysis in a patient with hyperthyroidism whose potassium level was within the normal range.

**Patient concerns:**

A 33-year-old Taiwanese man presented to the emergency department with bilateral limb weakness (more severe in the lower limbs than in the upper limbs). On arrival, the patient's vital status was stable with clear consciousness. He denied experiencing recent trauma, back pain, chest pain, abdominal pain, headache or dizziness, or a fever episode. Physical examination showed no specific findings. Neurological examination showed weakness in the muscles of the bilateral upper and lower limbs. Muscle weakness was more severe in the proximal site than in the distal site.

**Diagnosis:**

Blood examination showed normal complete blood count, normal renal and liver function, and normal potassium (3.5 mmol/L, normal range 3.5–5.1 mmol/L), sodium, and calcium levels; however, the examination showed impaired thyroid function (thyroid stimulating hormone: 0.04 uIU/mL, normal range 0.34–5.60 uIU/mL; free T4: 1.96 ng/dL, normal range 0.61–1.12 ng/dL). Brain computed tomography without contrast showed no obvious intra-cranial lesion.

**Interventions:**

Intravenous potassium infusion (20 mEq/L) with normal saline was prescribed for the patient.

**Outcomes:**

After treatment, the patient felt a decrease in limb weakness. He was discharged from our emergency department with a scheduled follow-up in the endocrine outpatient department.

**Lessons:**

TPP should be considered as a differential diagnosis in young Asian men presenting with limb paralysis that is more severe in the proximal site and in the lower limbs than in the distal site and in the upper limbs, respectively. It is important for emergency department physicians to consider TPP as a differential diagnosis as it can occur even if the patient's potassium level is within the normal range.

## 1 Introduction

Thyrotoxic periodic paralysis (TPP) is characterized by a sudden onset of hypokalemia and paralysis.^[1]^ The condition mainly affects the lower extremities and is secondary to thyrotoxicosis. The underlying hyperthyroidism is often subtle without typical symptoms such as palpitations, tremors, anxiety, and weight loss; this causes a difficulty in early diagnosis.^[2]^ Here, we reported a case of periodic paralysis in a patient with hyperthyroidism whose potassium level was within the normal range. The institutional review board of the Kaohsiung Medical University Hospital waived the need for ethical approval for this case report. Informed consent was obtained from the patient.

## 2 Case presentation

A 33-year-old Taiwanese man presented to the emergency department with bilateral limb weakness (more severe in the lower limbs than in the upper limbs). The patient's vital status on arrival was as follows: blood pressure, 160/86 mmHg; heartbeat, 96 bpm; and, body temperature, 36.3 °C, with clear consciousness. He denied experiencing recent trauma, back pain, chest pain, abdominal pain, headache or dizziness, or a fever episode. Physical examination showed no specific findings. Neurological examination showed weakness in the muscles of the bilateral upper limbs (muscle power score 4) and of the bilateral lower limbs (muscle power score 3). Muscle weakness was more severe in the proximal site than in the distal site. Blood examination included complete blood count, tests for renal and liver function, measurement of electrolyte levels (including potassium, sodium, and calcium levels), and tests for thyroid function. Blood examination showed normal complete blood count, normal renal and liver function, and normal potassium (3.5 mmol/L, normal range 3.5–5.1 mmol/L), sodium, and calcium levels; however, the examination showed impaired thyroid function (thyroid stimulating hormone: 0.04 uIU/mL, normal range 0.34–5.60 uIU/mL; free T4: 1.96 ng/dL, normal range 0.61–1.12 ng/dL). A 12-lead electrocardiogram showed normal sinus rhythm. Brain computed tomography without contrast showed no obvious intra-cranial lesion. Intravenous potassium infusion (20 mEq/L) with normal saline was prescribed for the patient. After the treatment, he felt a decrease in limb weakness. The clinical diagnosis favored TPP. This diagnosis was based on the clinical condition (bilateral muscle weakness that was more severe in the proximal site than in the distal site), age and race (33 years, Asian), family history (no history of periodic paralysis), blood examination results (hyperthyroid status but no obvious signs or symptoms of hyperthyroidism such as tremors, palpitation, or body weight loss), and imaging findings (brain computed tomography showed no obvious lesion); all these conditions matched the characteristics of TPP. The patient was discharged from our emergency department with a scheduled follow-up in the endocrine outpatient department (follow-up period of approximately 2 months). Methimazole (5 mg 3 times per day orally) was prescribed for controlling hyperthyroidism. He did not have any paralytic attack after discharge from our emergency department. Follow-up blood examination showed the following results: thyroglobulin antibody, <20 IU/mL and microsomal antibody, 36.9 IU/mL. Thyroid ultrasonography performed in the outpatient department showed no obvious mass or goiter.

## 3 Discussion

TPP is more commonly reported in the Oriental Asian population. Previous literature showed that the incidence of TPP in China and Japan was 1.8% and 1.9%, respectively; in contrast, the incidence was 0.1% to 0.2% in North Americans. In the Chinese population, TPP occurs in 13% of men and 0.17% of women thyrotoxic patients, according to a series published in 1967.^[3]^

The pathogenesis of TPP remains unclear. The sodium, chloride, calcium, and potassium channels on cell membranes are responsible for membrane excitability and muscle contractions. Disruption of any of these cellular transport mechanisms, especially the 3Na^+^/2K^+^ ATPase pump, may cause abnormalities in muscle contractibility, and paralysis. Recent studies have shown that mutations in the gene encoding Kir2.6, a skeletal muscle-specific Kir channel protein, are associated with TPP and predispose these individuals to acute paralytic attacks.^[1]^

The age of onset of TPP symptoms is mostly between 20 and 39 years. The paralytic attack is characterized by recurrent, transient episodes of muscle weakness that range from mild weakness to complete flaccid paralysis. Neurological examination during an attack demonstrates weakness, more commonly in the proximal muscles than in the distal muscles; furthermore, weakness is more common in the legs than in the arms.^[1]^ A patient with severe TPP and respiratory failure may need ventilator support. Arrhythmia can also be observed in TPP, and some reports have shown that severe and even fatal arrhythmia such as sinus arrest, atrioventricular block, ventricular tachycardia, and ventricular fibrillation can accompany TPP.

TPP must be distinguished from hypokalemic periodic paralysis, which is familial, more common in Caucasians, and is not associated with hyperthyroidism.^[1]^ Other causes of hypokalemia or proximal muscle weakness must be considered. Detection of hypokalemia in a patient and subsequent recovery with treatment generally alerts the clinician to a diagnosis of hypokalemic periodic paralysis; in such a case, the possibility of thyrotoxicosis must always be considered, particularly in a patient with no family history of periodic paralysis. The degree of hypokalemia during a TPP attack is variable; in 1 series of 78 patients, the mean serum potassium level was 2.1 mmol/L.^[4]^ Cases involving extremely low potassium levels (<1.5 mEq/L) are frequently reported. However, a case of periodic paralysis attributed to TPP, in which the patient's potassium level was normal has also been reported.^[5]^ Usually, the severity of muscle weakness corresponds to the degree of hypokalemia.

Treatment of TPP includes correction of hypokalemia and hyperthyroid status. Rebound hyperkalemia should be noticed during potassium supplementation. In 1 study, 80% of patients who developed rebound hyperkalemia had received >90 mEq of potassium within 24 hours.^[6]^

In the present case, the patient was a Taiwanese man aged 33 years. There was no prior event of a paralytic attack and family had no history of periodic paralysis. Neurological examination showed muscle weakness in the upper and lower limbs; the weakness was more severe in the proximal site than in the distal site, as well as more severe in the lower limbs than in the upper limbs. Blood examination showed an increase in free T4 level and a decrease in thyroid stimulating hormone level; therefore, hyperthyroidism was suspected, but the patient did not have any other symptoms characteristic of hyperthyroidism such as palpitations and body weight loss. The patient's potassium level was 3.5 mmol/L, which is on the lower side of the normal range. The clinical symptom of limb weakness subsided after the patient received 20 mEq of potassium intravenously. Although rare, paralysis occurred even when potassium level was within the normal range in our patient, who also had hyperthyroidism.

## 4 Conclusion

TPP should be considered as a differential diagnosis in young Asian men presenting with limb paralysis that is more severe in the proximal site and in the lower limbs than in the distal site and in the upper limbs, respectively. It is important for emergency department physicians to consider TPP as a differential diagnosis as it can occur even if the patient's potassium level is within the normal range.

## Author contributions

**Resources:** Yen-Hung Wu.

**Supervision:** Kuan-Ting Liu, I-Jeng Yeh.

**Visualization:** Yen-Hung Wu, I-Jeng Yeh.

**Writing – original draft:** Pin-Han Wang, Yen-Hung Wu.

**Writing – review & editing:** Kuan-Ting Liu.
